# Pain in Children and Adolescents with Spinal Muscular Atrophy: A Longitudinal Study from a Patient Registry

**DOI:** 10.3390/children10121880

**Published:** 2023-11-30

**Authors:** Inmaculada Pitarch-Castellano, David Hervás, Maria Grazia Cattinari, Eugenia Ibáñez Albert, Mercedes López Lobato, Nancy Carolina Ñungo Garzón, Juan Rojas, Cristina Puig-Ram, Marcos Madruga-Garrido

**Affiliations:** 1 Neuropediatric Department, Hospital Universitario y Politécnico la Fe, 46026 Valencia, Spain; pitarch_inmcas@gva.es; 2 Department of Applied Statistics and Operations Research and Quality, Universitat Politècnica de València, 46022 Valencia, Spain; daherma@eio.upv.es; 3 Fundación de Atrofia Muscular Espinal, FundAME, 28034 Madrid, Spain; 4 Rehabilitation Department, Hospital Universitario y Politécnico la Fe, 46026 Valencia, Spain; 5 Neuropediatric Department, Hospital Universitario Virgen del Rocío, 41013 Sevilla, Spain; 6 Neuromuscular Diseases Unit, Hospital Universitario y Politécnico la Fe, Institute for Health Research La Fe (IISLAFE), 46026 Valencia, Spain; carolina_nungo@iislafe.es; 7 Faculty of Medicine, Universitat de València, 46010 Valencia, Spain; 8 Rehabilitation Department, Hospital Universitario Virgen del Rocío, 41013 Sevilla, Spain; 9 Neuromuscular Diseases Unit, Institut de Recerca Sant Joan de Déu, Santa Rosa 39-57, 08950 Esplugues de Llobregat, Spain; 10 Sección de Neurología Pediátrica, Hospital Universitario Virgen del Rocío, 41013 Sevilla, Spain

**Keywords:** pain, spinal muscular atrophy, neuromuscular disease, children, adolescent natural history, patient registry, real-world evidence

## Abstract

Spinal muscular atrophy (SMA) is a devastating genetic neurodegenerative disease caused by the insufficient production of Survival Motor Neuron (SMN) protein. It presents different phenotypes with frequent contractures and dislocations, scoliosis, and pain. This study aims to report the prevalence and description of pain and how it affects daily life by analyzing a new ad hoc questionnaire. An observational study of patients under 18 years of age with SMA was conducted at two referral centers in Spain. Data were analyzed using a descriptive analysis and a Bayesian ordinal regression model to assess the association with clinical and demographic variables. Fifty-one individuals were included in this study, 27% of whom reported pain with a median duration of 5.2 years and a mean Visual Analogic Scale (VAS) score of 5. Notably, 77% were receiving disease-modifying treatment, with more than 50% receiving analgesic treatment. The Bayesian model showed that functional status, lower limb contractures, and number of visits have a high probability (>90%) of influencing pain. Thus, the prevalence of pain in the SMA population under 18 years is substantial, and its presence could be associated with lower limb contractures, better functional status, and higher RULM (Revised Upper Limb Module) scores.

## 1. Introduction

Spinal muscular atrophy (SMA) is an autosomal recessive neuromuscular disorder caused by the degeneration of alpha motor neurons of the anterior horns of the spinal cord, resulting in lower Survival Motor Neuron (SMN) protein production and leading to progressive muscle atrophy and weakness [1,2], making it a devastating neurodegenerative disease [3]. The severity of SMA is highly variable, and clinical features have been classically classified into phenotypes based on the age of onset and maximum motor function achieved: type 0 or the prenatal type; very weak infants unable to sit unsupported (type I); non-ambulant patients able to sit independently (typically type II); up to ambulant patients during childhood who generally lose the ability to walk in later years (type III); and adult-onset SMA (type IV) [2,4,5,6]. In particular, type II and III SMA patients frequently present contractures of the lower extremities (with hip subluxation and dislocations. It has been documented that scoliosis emerges in nearly 100% of non-ambulatory SMA patients, who present early and with a severe progression [7]. All of these causes and the orthopedic procedures employed to manage them could produce pain that affects their quality of life (QoL) [8,9,10].

There is a wide selection of QoL-measuring tools available in the literature for populations with neuromuscular disorders; however, they are only relevant in specific populations and disease areas [11,12,13,14] Some studies have been carried out using different tools and questionnaires to measure pain. Initial studies investigated adult subjects with neuromuscular disorders using different tools, such as the Medical Outcomes Study SF-36 health survey, the Neuropathic Pain Scale, or Brief Pain Inventory (BPI), concluding that these disorders present with significant pain and distinguishing the nature of the pain and the impact among neuromuscular diagnostic groups [15,16]. Afterward, a publication delved deeper into the prevalence and characteristics of pain in children with neuromuscular disease using a numerical rating scale and a modified BPI, highlighting the prevalence and chronicity of pain in youths with neuromuscular diseases [17]. Recently, a study showed that 55% of adult SMA type III walker patients in their sample presented nociceptive pain, as measured with different tools (pressure algometer, myotonometry), hypothesizing that it is caused by changes in the imbalanced musculoskeletal system due to muscle weakness [18]. Finally, another recent study in SMA patients, both children and type II and type III adults, using a questionnaire of in-house design, showed chronic, frequent, and low-intensity pain among the SMA population [19]. Therefore, well-designed patient and caregiver-oriented outcome measures are still needed to ensure that we are measuring the most relevant and clinically meaningful outcomes [20]. Since 2017, the landscape in SMA has been promising, with improvements in motor and respiratory function and nutrition due to disease-modifying treatments [21,22,23,24,25,26,27] and updated standards of care [8,28]. Thus, it is time for a more extensive investigation of the QoL field, and, taking into consideration the prevalence of pain in the SMA population, studies are needed to help assess the effectiveness of and further inform treatment algorithms [28].

This paper aims to study the existence of pain in a large sample of pediatric patients with SMA and to characterize disease progression by describing how pain affects their daily lives via a preliminary analysis of a new ad hoc questionnaire.

## 2. Materials and Methods

This was a three-year prospective observational follow-up study of routine practice that aimed to determine the progress of children and adolescents with SMA conducted between April 2017 and May 2020 by pediatric neurologists at two sites. The main objective of this study was to determine the natural history of the development of SMA in the Spanish population; this was achieved by using the measurement of changes in functional levels through validated motor scales appropriate for each patient according to their age and type of SMA. Additionally, this study outlined specific goals: defining the fundamental attributes of the study population, characterizing disease progression whilst stratifying it into subgroups, and ascertaining the influence of disease progression on the quality of life of individuals with SMA, among others.

The database contained clinical data collected during routine visits, such as the age of symptom onset, *SMN2* gene copy number, maximum achieved motor function, respiratory status, digestive and nutritional status, support for rehabilitation and having had scoliosis surgery, hospitalizations, and orthopedic status, including the presence of pain or fatigue. In the case of presenting pain, the presence, localization, or severity were ascertained. Motor scale scores were also recorded as a current tool to measure motor ability and clinical development, including the Revised Upper Limb Module (RULM), the Hammersmith Functional Motor Scale Extended (HFMSE), the Children’s Hospital of Philadelphia Infant Test of Neuromuscular Disorders (CHOP INTEND), the Hammersmith Infant Neurological Examination Section 2 (HINE-2), the 6-Minute Walk Test (6MWT), and the Egen Klassifikation (EK 2). Finally, patient-reported outcomes related to pain were also recorded. The HFMSE consists of a validated scale for SMA type II or III individuals with limited mobility despite their functional status [29,30]. The RULM scale is a tool used to assess upper extremity function in the SMA population [31]. Both scales offer clinicians a sensitive and robust clinical measure and are extensively used in clinical practice and also for investigational purposes [8].

The pain questionnaire was developed considering the opinion of patient experts and clinicians specializing in the disease and contains twelve questions about localization, intensity, duration, treatment, and a visual analog scale (VAS) ranging from 0 to 10 or images for the youngest patients. The questionnaire also included questions regarding how pain affected their day-to-day activities (see Table 1 for the full questionnaire).

The data were collected by clinical specialists from both participating hospitals every six months and uploaded to a platform owned by FundAME (the Spanish SMA Family Organization); in addition, patients or caregivers reported data such as pain and QoL via a study questionnaire. Data curation was performed by a physician expert in SMA. The registry Information Technology (IT) platform was hosted on Azure, a suite of cloud services owned by Microsoft, with proprietary source code. The server was safeguarded by passwords, additional security measures, and Azure’s security infrastructure.

In this study, we included all responses from SMA patients under 18 years of age and with a genetically confirmed diagnosis (a 5q13 chromosome alteration or homozygous deletion or mutation of the *SMN1* gene). Due to the observational nature of this study, no formal sample size was performed. However, we planned to include data on identified patients with SMA during follow-up in the participating centers, as well as data on new patients diagnosed during the first year of the follow-up period.

Ethics committee approval was acquired by the participating centers prior to the initiation of this study (Hospital Universitario y Politécnico La FE in Valencia (FUN-000-2017-01) and Hospital Universitario Virgen del Rocío in Sevilla (12/2017)), and written informed consent was obtained from all patients or legal representatives.

### Statistical Analysis

Data are presented as mean (standard deviation) and median (1st, 3rd quartile) in the case of continuous variables and as absolute (relative) frequencies in the case of categorical variables. To assess the association between pain and the different clinical and demographic variables, a Bayesian ordinal regression model was adjusted. A random intercept and random slope for visit number were added to account for the non-independence of observations. Additional models were adjusted to assess trends in the RULM and HFMSE scales and their association with pain. A 95% highest density credibility interval was estimated for each parameter in the models, as well as the probability of each parameter being greater or lower than zero. Descriptive analysis and other analyses were performed using R (version 4.2.3) and the R packages brms (version 2.18) and bayestestR (version 0.13).

## 3. Results

Data from one to four visits for 51 individuals were included in this study. Table 2 summarizes the most relevant characteristics of the participants enrolled in this study. In our study, 43% (*n* = 22) of patients reported pain with a mean VAS of 5 (range: 2–10) and a median duration of 5.2 years (ranging from 0 to 10 years). Among those experiencing pain, 45% (*n* = 10) reported pain at different locations simultaneously. Of note, 77% (*n* = 18) of individuals who suffered from pain were receiving a disease-modifying therapy (DMT).

Focusing on specific body locations, our study found that 32% (*n* = 7) of participants experienced hip pain with a moderate level of intensity (mean VAS of 5.3). Of these individuals, three further described this pain as the most debilitating. Among the 45% (*n* = 10) of participants who reported back pain (mean VAS of 5.1), 6 reported this pain to be the most incapacitating. Finally, 82% (*n* = 18) of those surveyed described pain sensations in other locations with an average VAS of 4.7, with the majority (*n* = 15) of them also considering this pain to be the most disabling. Some patients reported limitations because of the pain, such as difficulty attending school (*n* = 3), sleep disruption (*n* = 3), or pain at rest (*n* = 8). More than 50% of the population were treated to alleviate pain using analgesia (35%), 53% received physiotherapy, and 12% were treated with other methods.

Since data from different visits were collected for each patient, a longitudinal analysis of the different variables was performed. The Bayesian ordinal regression model used to explain pain values found that higher functional levels, lower limb contractures, and high visit numbers have a high probability (>90%) of influencing pain as measured with VAS scores. The model also exhibited moderately high evidence regarding the influence of age (84.5% probability); other factors had a much lower probability. All results are presented in Table 3.

**Figure 1:**
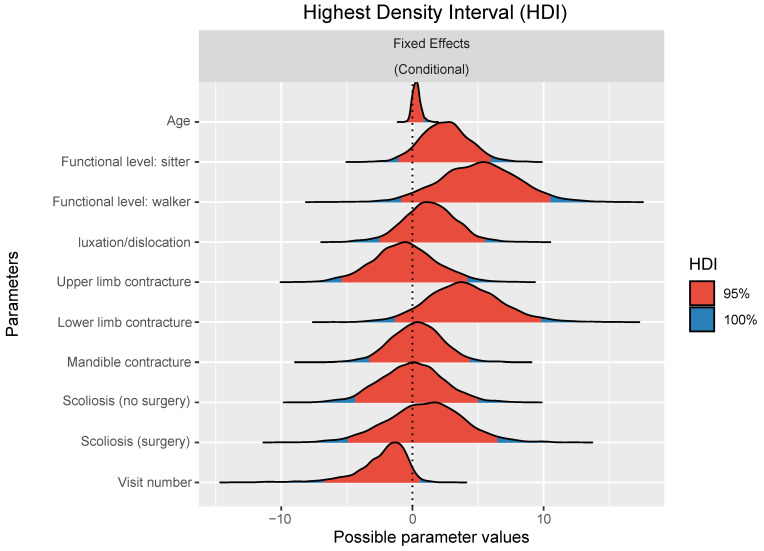
Highest Density Interval (HDI).

A plot of the estimated posterior distributions for the effect of each variable is presented in Figure 1.

RULM scale analysis showed that increased scores on this scale over time are directly related to an increase in pain VAS scores (96.05% probability). Conversely, in the case of HFMSE, the probability of an association with pain VAS scores was lower (76% probability). Detailed results from these models are available in Table 4 and Table 5.

## 4. Discussion

The results of this study show that pain in children and adolescents with SMA is a worrisome issue, being mainly associated with a better functional status and higher RULM scores, as well as the existence of contractures of the lower extremities. On the other hand, a higher number of visits is associated with a lower likelihood of pain. These results contribute to improving the knowledge of pain in children with SMA.

Only one of the aforementioned studies [15,16,17,18,19,34] reported results in a pediatric SMA population. The results of Lager et al.’s study on 17 teenagers with SMA highlighted that pain is a frequent problem in adolescents, with 69% of the population reporting pain within the last three months and 50% reporting chronic pain [34]. Similarly, Uchio et al. [16] detected chronic pain in 40% of patients with SMA types II and III in a mixed adult and pediatric population, and a recent study on nociceptive pain in adult SMA type III walker patients found that 55% of the population reported pain [18]. In our pediatric cohort, similar to the previously mentioned publications, 43% of the population suffered from pain, with a mean age of eight years and different SMA types, and almost one in three patients had chronic and, in some cases, disabling pain at different locations; therefore, more than half of the affected participants might be treated with analgesia, physiotherapy, or other methods to alleviate pain symptoms.

On the other hand, to our knowledge, this is the first publication correlating pain with other variables such as age, functional level, the presence of contractures (located in upper limbs, lower limbs, jaw) or dislocations, scoliosis surgery, and number of visits. Hence, walker status or higher RULM scores have a strong association with higher VAS scores. Similarly, sitter status also correlates with the presence of pain compared with non-sitters. However, this study did not have sufficient information to produce conclusive results regarding the HFMSE scale, probably due to the reduced sample size. Other scales were not taken into account in the model, mainly because the other scales were not age-appropriate or the sample of patients was not sufficient to draw conclusions from the results. Taken together, it seems that patients with a better functional status are more likely to suffer from pain. These results should be interpreted with caution; nevertheless, they agree with the hypothesis that muscle overuse may be a potential cause of pain, as mentioned in a study that identified excessive movement/activity as a factor exacerbating pain in a cohort of adolescents with neuromuscular disorders, especially in walkers [34]. In addition, the study on nociceptive pain in adult SMA type III walker patients concluded that their pain was associated with an imbalance of the musculoskeletal system due to muscle weakness [18]. Hence, the prevalent idea of extending the intervals for patient follow-up when they are in good condition should be discarded, as these patients are actually more susceptible to experiencing pain.

Furthermore, in our study, age seemed to have a moderate association with pain, while another study produced different results [35]. These differences are probably because our sample comprised patients under 18, while the previous study focused on adults.

Regarding hip pain, it was reported that 49% of SMA type II children present hip pain [36], which increased to 58% in a telephone survey of a wider SMA population, which showed moderate-to-severe pain in 14% of the population [37]. Interestingly, in contrast to the common belief that luxation is not painful, our study showed that luxation might increase the pain VAS score (75% probability). In our series, hip pain was recorded in nearly one out of every three patients. Even though pain due to hip luxation and dislocation is a cause for concern, its management is controversial [36,37]. Our study highlights the need to pay more attention to luxation/dislocations, and further investigation in this field is necessary. Notwithstanding, the association between the spine and hip pain in patients with neuromuscular conditions has already been established [38]. Previous reports found no relation between patients with scoliosis and hip pain requiring invasive treatment [36,39]; similarly, we did not find a high level of association between a history of scoliosis surgery and no history. It may be that, in this case, the sample is biased since, in general, only severe cases of scoliosis require surgical intervention; also, the time elapsed since surgery was not analyzed.

Additionally, it is well known that limb contractures are a common impairment in SMA, and some reports studied their relation to motor performance and mobility limitations [9,40]. However, we found scant updated literature on the association between lower limb contractures and pain in the disease. Therefore, this study shows that there is an association between pain and contractures of the lower extremities. Of the variables identified in this analysis as having a high likelihood of influencing pain, only limb contractures appeared to be addressed proactively. Early diagnosis and initiation of physical medical approaches, such as passive range of motion and splinting before contractures occur or while contractures are mild, seem to be good options to minimize the impact or disability of contractures [41]. On the contrary, jaw and upper limb contractures had a low level of association, although this association was reported in the literature some time ago [42].

In parallel, our study identified a higher number of visits as a variable strongly associated with a reduced likelihood of pain. It can be hypothesized that a greater number of visits implies longer and closer monitoring of the patient and a greater impact on interventions [8]. Moreover, patients’ adherence to visits should be considered a factor in their pain control.

Furthermore, our study found that pediatric SMA patients present chronic pain (mean duration 5.2 years) at different locations. It is well known that pain has a direct impact on QoL [13], reflecting that the pediatric SMA patients in this study experienced a negative impact on their QoL.

Despite advances in SMA management, no standard tool has been identified to adequately track pain in this disease. Hence, specific patient-reported outcome measures (PROMS) that actually measure the needs of SMA patients and with patient involvement in the design are needed. Meanwhile, we employed a questionnaire designed by patient experts to reflect those variables with the greatest impact on pain in SMA patients (see the questionnaire in Table 1). This practice has limitations as, in some items, the pain reported by young SMA patients was based on their parent’s judgment, despite the Numeric Rating Scale (NRS) being carried by the patient; therefore, parental opinions could introduce bias into some answers.

Finally, we recognize that we do not know the effect of some variables that were not included in our model, such as psychological state, gender, effect of concomitant medication, respiratory status, etc. Additional information would add context and broaden the interpretation of our results.

## 5. Conclusions

Our results demonstrate that the prevalence of pain in the pediatric SMA population is considerable; furthermore, its presence could be correlated with lower limb contractures, better functional status, and higher RULM scores. Overall, closer monitoring of dislocations and proactive management of contractures could have an impact on reducing pain and improving the QoL of patients and caregivers, particularly in the ambulant population.

## Figures and Tables

**Table 1:** Patient questionnaire to assess pain.

1. Are you experiencing hip pain? Yes/No
2. Are you experiencing back pain? Yes/No
3. Are you experiencing pain in other musculoskeletal localizations? Yes/No
i. Please specify
4. If more than one “yes” response was selected above, please choose the most painful or disabling pain
5. Focusing on the most disabling pain:
i. Starting age (months or years, please specify)
ii. VAS (Visual analog scale) score from 0 to 10
6. Are you experiencing resting pain? Yes/No
7. Are you experiencing pain at manipulation? Yes/No
8. Does pain disrupt your sleep? Yes/No
9. Are you experiencing other kinds of pain? Please specify
10. Does pain impact your work or schoolwork? (Performance or attendance) Yes/No
11. Are you experiencing pain when standing? Yes/No
12. Do you receive pain treatment? Please select: analgesia, physiotherapy, or others
i. If “others” is selected, please specify

Note: the original questionnaire was written in Spanish as it is the mother tongue of the participants and has been translated for the purposes of the article.

**Table 2:** Baseline characteristics of the included patients.

Variable	N = 51 Mean (SD)/n (%) Median (1st, 3rd Q)
Current age (years)	8.49 (5.33)
	8 (4, 13.5)
Age at onset of symptoms (months)	10.78 (8.48)
	8 (6, 15)
Age at diagnosis (months)	22.25 (23.24)
	15 (10.5, 24)
Gestational age (weeks)	39.23 (1.6)
	40 (39, 40)
Sex assigned at birth	
Male	26 (50.98%)
Female	25 (49.02%)
SMA type	
Type I	13 (25.49%)
Type II	24 (47.06%)
Type III	14 (27.45%)
Functional status ^1^	
Non-sitter	19 (37.25%)
Sitter	20 (39.22%)
Walker	12 (23.53%)
Genetic confirmation	
Yes	51 (100%)
Genetic study	
Deletion of the 2 copies of *SMN1*	49 (96.08%)
Deletion of 1 copy and point mutation on the other copy of *SMN*1	1 (1.96%)
Deletion of 1 copy of *SMN1*/point mutation, unknown mutation on the other copy	1 (1.96%)
No. of *SMN2* copies	
2	16 (31.37%)
3	29 (56.86%)
4	5 (9.8%)
unknown	1 (1.96%)
Prenatal symptoms onset	
no	50 (98.04%)
yes	1 (1.96%)
Newborn screening	
no	51 (100%)
Family history of SMA	
no	44 (86.27%)
yes	7 (13.73%)

SD: standard deviation; Q: quartile; SMA: Spinal Muscular Atrophy; SMN: Survival Motor Neuron. Note: ^1^ Functional status is classified using [32,33]. Sitter: can sit without support; walker: can walk 10 m without assistance.

**Table 3:** Results of the ordinal regression model for pain measured by VAS scores.

Variables	Estimate	Std. Error	OR	95% CI	*p* (Effect > 0)	*p* (Effect < 0)
Age	0.303	0.308	1.354	[0.73, 2.38]	84.45%	15.55%
Functional level: sitter	2.503	1.816	12.218	[0.34, 407.72]	92.38%	7.62%
Functional level: walker	5.095	2.96	163.273	[0.42, 37,452.3]	95.75%	4.25%
Luxation/dislocation	1.305	2.006	3.689	[0.08, 251.76]	75.30%	24.70%
Upper limb contracture	−0.583	2.414	0.558	[0.004, 65.93]	38.75%	61.25%
Lower limb contracture	3.963	2.812	52.635	[0.27, 18676.0]	92.95%	7.05%
Mandible contracture	0.33	1.954	1.391	[0.04, 80.77]	57.20%	42.80%
Scoliosis (no surgery)	−0.028	2.391	0.973	[0.01, 149.51]	49.95%	50.05%
Scoliosis (surgery)	0.987	2.89	2.684	[0.007, 646.10]	64.28%	35.72%
Visit number	−2.313	1.998	0.099	[0.001, 1.76]	4.55%	95.45%

VAS: visual analog scale; Std: standard; CI: Confidence Interval; OR: Odds Ratio.

**Table 4:** Model for RULM scale.

Variables	Estimate	Std. Error	OR	Lower Bound CI.	Upper Bound CI
Age	−0.185	0.138	0.832	0.629	1.085
Functional level: sitter	2.019	1.279	7.529	0.646	92.311
Functional level: walker	5.475	1.905	238.56	6.352	11,167.969
Lower limb contracture	−1.618	1.5	0.198	0.009	3.561
Visit number	−0.389	0.525	0.678	0.209	1.732

RULM: Revised Upper Limb Module; Std.: standard; OR: Odds Ratio; CI: Confidence Interval.

**Table 5:** Model for HFMSE scale.

Variables	Estimate	Std. Error	OR	Lower Bound CI	Upper Bound CI
Age	−0.231	0.14	0.794	0.582	1.028
Functional level: sitter	2.768	1.559	15.928	0.824	407.254
Functional level: walker	8.357	2.112	4261.209	78.768	353,131.089
Lower limb contracture	−1.467	1.452	0.231	0.01	3.87
Visit number	0.032	0.439	1.033	0.432	2.578

HFMSE: Hammersmith Functional Motor Scale Extended; Std.: standard; OR: Odds Ratio; CI: Confidence Interval.

## Data Availability

The datasets generated and/or analyzed during the current study are not publicly available due to ethics restrictions but are available from the corresponding author on reasonable request.
